# Phosphorylation of hnRNP K controls cytosolic accumulation of TDP-43

**DOI:** 10.1186/1750-1326-8-S1-P46

**Published:** 2013-09-13

**Authors:** Anthony White, Diane Moujalled, Janine James, Alexandra Grubman, Katja Kanninen, Peter Crouch

**Affiliations:** 1 Department of Pathology, The University of Melbourne, Parkville, Australia; 2 Laboratory of Molecular Brain Research, A.I. Virtanen Institute for Molecular Sciences, University of Eastern Finland, Kuopio, Finland

## Background

TAR-DNA binding protein 43 (TDP-43) is a heterogeneous ribonucleoprotein (hnRNP) identified as a major protein constituent of cytosolic inclusions in spinal cord of patients with motor neuron disease. The inclusions are formed by movement of TDP-43 from its predominantly nuclear localization to the cytosol, followed by accumulation of ubiquitinated and phosphorylated C-terminal TDP-43 fragments. The mechanisms controlling TDP-43 trafficking and accumulation are not well known, however, it has been demonstrated previously that kinases control movement and accumulation of hnRNPs. Therefore, we investigated whether kinases also controlled TDP-43 accumulation during cell stress.

## Materials and methods

Neuronal cultures were treated with the mitochondrial inhibitors paraquat or sodium arsenite, or transfected with C-terminal TDP-43 or full length TDP-43 constructs and examined for kinase control of TDP-43 localization.

## Results

We found that stress induction induced robust cytosolic accumulation of C-terminal TDP-43 into RNA stress granules, some of which progress to ubiquitinated inclusions. Inhibitors of c-Jun N-terminal kinase (JNK) and cyclin-dependent kinase 2 (CDK2) specifically inhibit and/or reverse the cytosolic accumulation of TDP-43 with little effect on ubiquitous stress granule formation. Our studies have shown active CDK2 co-localizes with accumulated TDP-43 and heterogeneous ribonucleoprotein K (hnRNP K). CDK2 inhibition blocks phosphorylation of hnRNP K, preventing its incorporation into stress granules. Due to Interaction between hnRNP K with TDP-43, the loss of hnRNP K from stress granules prevents accumulation of TDP-43. We have also determined that specific C-terminal mutations to TDP-43 alter expression of hnRNP K during cell stress. Preliminary studies indicate that this may involve altered hnRNP K phosphorylation.

## Conclusions

Due to the recently identified role for hnRNPs in motor neuron disease and frontotemporal dementia, further investigation of the association between hnRNP K and TDP-43 is warranted. Understanding how kinase activity modulates TDP-43 accumulation may provide new pharmacological targets for disease intervention.

